# Expanded trade: tripartite interactions in the mycorrhizosphere

**DOI:** 10.1128/msystems.01352-23

**Published:** 2024-06-05

**Authors:** Christos Charakas, Devanshi Khokhani

**Affiliations:** 1 Department of Plant and Microbial Biology, University of Minnesota, Twin Cities, Minnesota, USA; 2 Department of Plant Pathology, University of Minnesota, Twin Cities, Minnesota, USA; University of California San Diego, La Jolla, California, USA

**Keywords:** arbuscular mycorrhizal fungi, tripartite interactions, mycorrhizosphere, nutrient exchange, compartmented systems, stable isotopes

## Abstract

Interactions between arbuscular mycorrhizal fungi (AMF), plants, and the soil microbial community have the potential to increase the availability and uptake of phosphorus (P) and nitrogen (N) in agricultural systems. Nutrient exchange between plant roots, AMF, and the adjacent soil microbes occurs at the interface between roots colonized by mycorrhizal fungi and soil, referred to as the mycorrhizosphere. Research on the P exchange focuses on plant–AMF or AMF–microbe interactions, lacking a holistic view of P exchange between the plants, AMF, and other microbes. Recently, N exchange at both interfaces revealed the synergistic role of AMF and bacterial community in N uptake by the host plant. Here, we highlight work carried out on each interface and build upon it by emphasizing research involving all members of the tripartite network. Both nutrient systems are challenging to study due to the complex chemical and biological nature of the mycorrhizosphere. We discuss some of the effective methods to identify important nutrient processes and the tripartite members involved in these processes. The extrapolation of *in vitro* studies into the field is often fraught with contradiction and noise. Therefore, we also suggest some approaches that can potentially bridge the gap between laboratory-generated data and their extrapolation to the field, improving the applicability and contextual relevance of data within the field of mycorrhizosphere interactions. Overall, we argue that the research community needs to adopt a holistic tripartite approach and that we have the means to increase the applicability and accuracy of *in vitro* data in the field.

## INTRODUCTION

Similar to the microbiota residing in the digestive tracts of vertebrates (1), microbes proliferating at the interface of plant roots and soil, also called the rhizosphere, can help improve plant health and agricultural productivity (2). Particularly, the interactions between plant roots, mycorrhizal fungi (obligate root endosymbionts), and the greater rhizospheric community (bacteria, archaea, protists, and viruses), also called the mycorrhizosphere, can increase nutrient availability in soil and its uptake by plants (2). The United Nations predicts the global population will increase to ~9.5 billion by 2050, requiring an ~ 70% increase in food production (3). A major limiting factor to agricultural productivity is plants’ ability to acquire and use soil nutrients, particularly phosphorus (P) and nitrogen (N). Although synthetic fertilizers increase available nutrients to boost crop yields (4), about 40%–60% of applied N and varying P amounts are often lost in agricultural runoff water (5–7). This runoff causes harmful algal blooms and pollutes groundwater (6, 8, 9). Furthermore, genetic and environmental factors also impact the plants’ ability to acquire applied N and P (5, 6, 10–13), making it difficult for growers to apply the right amount of fertilizers at the right time. To reduce further environmental damage, we need to adopt practices that can reduce the dependence on synthetic fertilizers. With decades of research on plant-associated microbes, we can harness the benefits of soil microbial relationships with plants to improve crop nutrient uptake.

The exemplary plant–microbe nutrient relationship involves N-fixing bacteria known as diazotrophs that provide a variety of crops with atmospherically derived N in forms accessible for plant uptake. Plants in the Fabaceae family, known as legumes, form a symbiotic relationship with root-nodulating diazotrophs such as as rhizobia to acquire N in the form of NH_3_/NH_4_^+^ in exchange for carbon (C) (14). Farmers use this relationship to provide crop rotations, hence reducing chemical N input (15). However, in case of non-legumes such as wheat, maize, and rice, non-rhizobial free-living (16) or root-associative (17) diazotrophs only loosely associate with roots and hence provide little N compared to rhizobia. Diazotrophs are also subject to tight feedback regulations of N-fixation, resulting in little to no ammonia excretion (18). Since cereal crops form a major fraction of human calorie intake compared to legumes (19), improving biological nitrogen fixation (BNF) in non-legumes is of great interest (20).

Over 70% of land–plant roots form symbiotic partnerships with obligate biotrophs called arbuscular mycorrhizal fungi (AMF), and about 13% of land plants are colonized by other mycorrhizal fungi, including ectomycorrhizal, ericoid, and orchid mycorrhizal fungi (21, 22). In this review, we focus on AMF interactions owing to their intimate relationship with a vast majority of land plants. Followed by biochemical exchanges, AMF invaginate their hyphae directly into cortical cells of plant roots, creating “arbuscules” where nutrient exchanges occur (21). AMF transport P (23–25) and N (26–29) to the plant in exchange for resources, usually C (27, 30). With limited ability to access and process organic forms of P and N (31, 32), AMF rely on other soil microbes to free up these valuable resources in a different focal environment called the “mycorrhizosphere” (27, 33, 34), a complex nexus of plant roots, extraradical hyphae of the mycorrhizal fungi, and other microbes in the rhizosphere.

**Fig 1:**
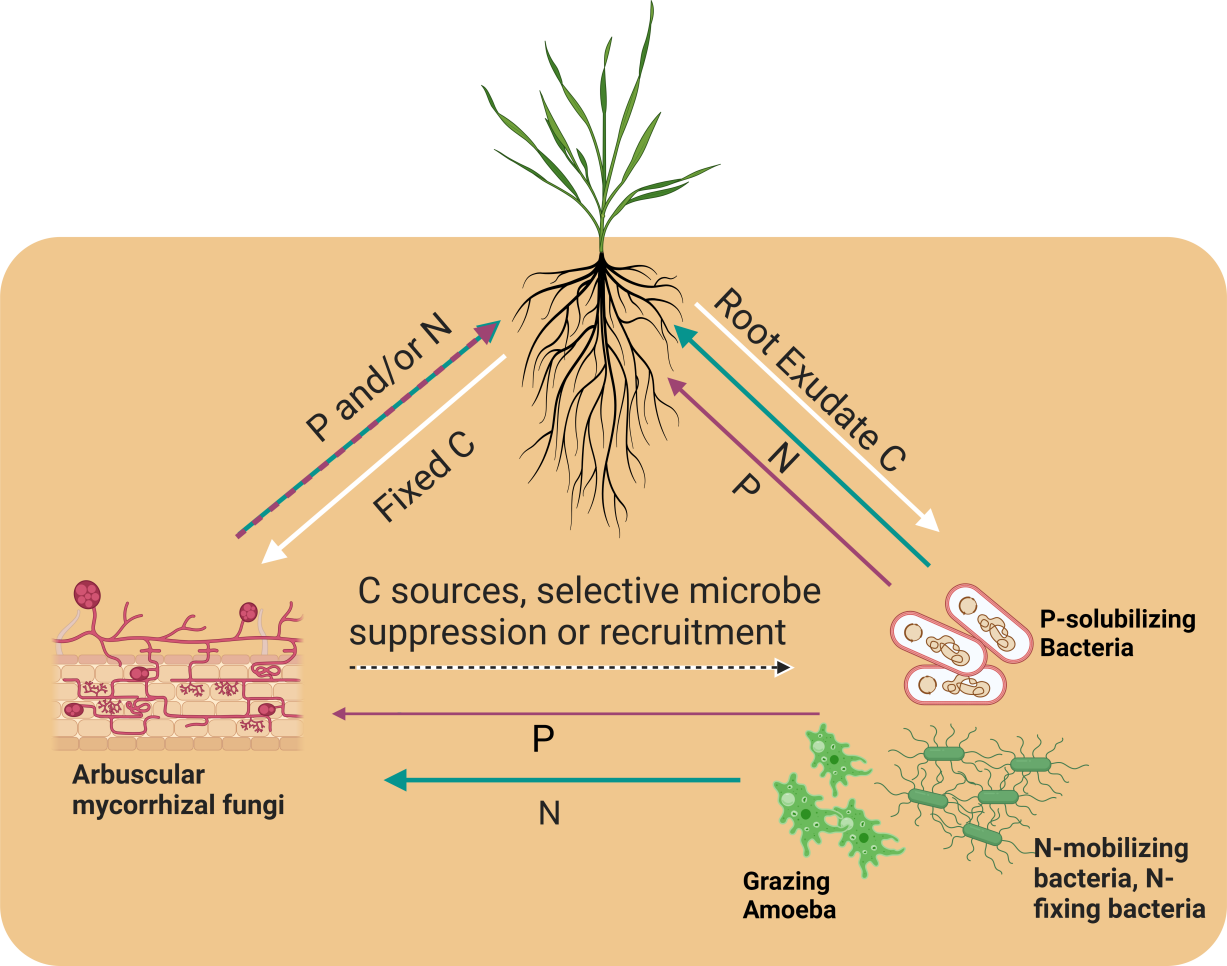
Tripartite nutrient interactions in the mycorrhizosphere. Depicted are the carbon (C), nitrogen (N), and phosphorus (P) sources and sinks. White lines represent C, blue-green lines represent N, magenta lines represent P, and dashed lines represent one organism performing multiple processes. Figure was created using BioRender.

This review looks at the mycorrhizosphere through the perspective of tripartite interactions between plants, AMF, and the microbial community (Fig. 1). While there is extensive research on nutrient dynamics at the AMF–plant interface (35) and relatively less at the interface of the AMF and soil microbial community (36), these research perspectives focus on two separate interfaces of a whole system. Here, we will consider these different interfaces simultaneously and review research that has focused on this wider perspective. Finally, we will discuss methods that could provide a more comprehensive understanding of the tripartite network. We aim to reveal an interconnected system of nutrient exchange among plants, AMF, and microbes in order to present these tripartite interactions holistically for a better understanding of nutrient exchange.

## DIVERSIFIED TRADE

### Phosphorus

While plants can acquire inorganic and organic P in close proximity to roots, hyphae of AMF can extend into the soil far beyond the root surface and access inorganic P located much farther from the plant (37, 38). AMF’s successful colonization of roots is dependent on soil P levels, cementing P as an important factor in the establishment and maintenance of symbiosis (39). AMF allocate inorganic P to the most advantageous places with precision (25, 40, 41) by providing more P to newly forming lateral roots that offer more C to the AMF due to their more immediate need for P (41). We now understand that AMF-sourced inorganic P in plants depends on the photosynthate provided to AMF, suggesting that control of P flow is mediated by a C price (25). This so-called “exchange rate” has favored evolutionary fitness-enhancing strategies in both organisms (Fig. 1).

Importantly, AMF do not mobilize organic P but recruit and interact with soil bacteria, creating a tripartite system involving nutrient trade (34). Phosphorus-solubilizing bacteria (PSB) help break down P-rich and chemically complex phytate in optimum P conditions, allowing for an increase in plant-shoot P when bacteria and AMF are present together, relative to AMF or bacteria alone (34). This suggests that AMF can acquire bacterially solubilized P and transfer it to the plant. However, this interaction is complex and qualitatively dependent on inorganic P in the soil (34). Fructose exuded from extraradical hyphae induces the expression of phosphatases and P transporters in the PSB, leading to phytate mineralization (42). This induction suggests that hyphal exudates act as a cue to initiate P acquisition from bacteria, presenting a possible inverse relationship between the PSB and AMF. The plant–AMF nutrient exchange would then be influenced by the AMF–PSB nutrient exchange (Fig. 1). Research on P and C allocation and exchange strategies at the AMF–PSB and AMF–plant interfaces simultaneously would help characterize the nutrient value and fitness dynamics in the system.

### Nitrogen

**Fig 2:**
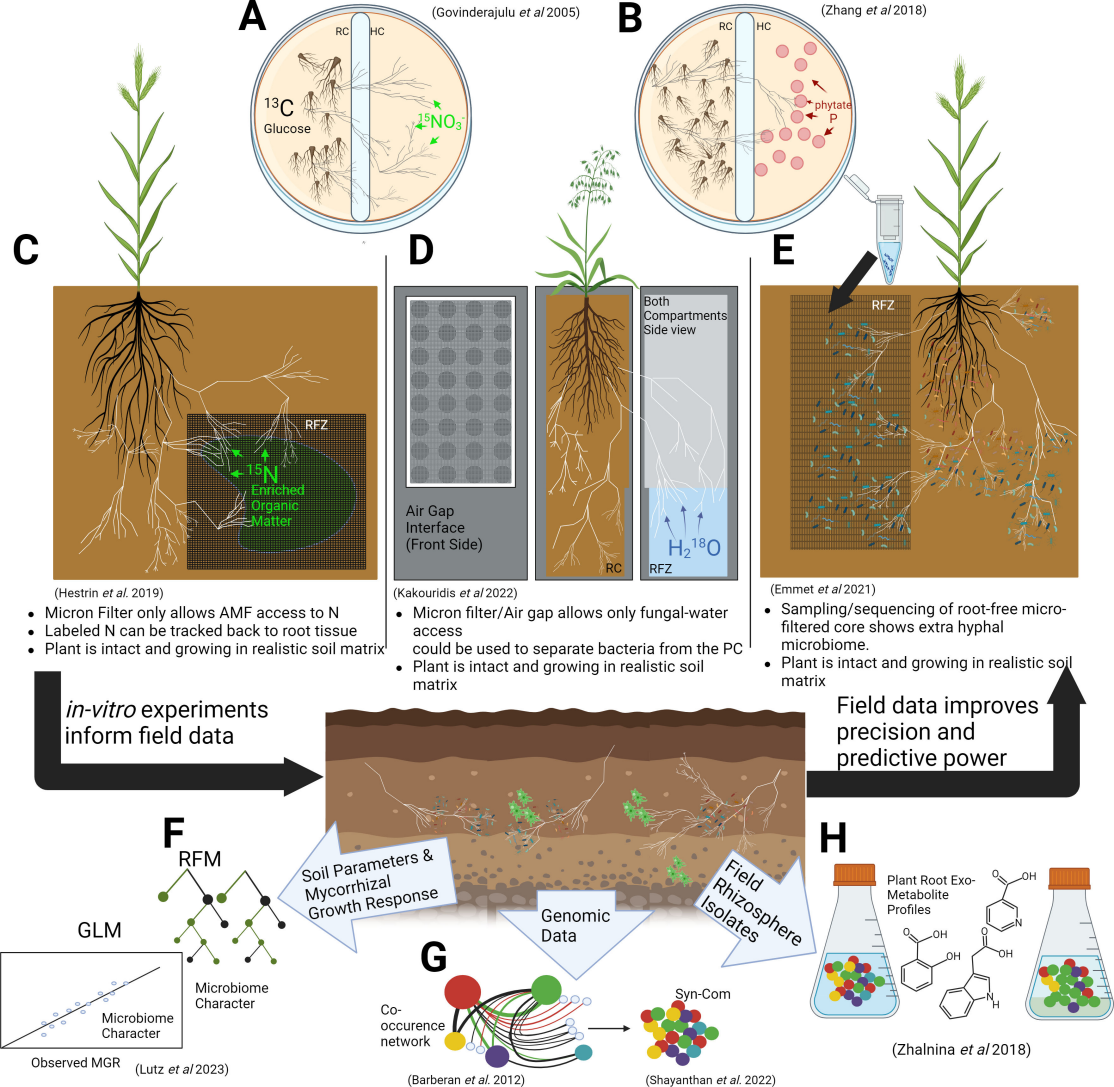
Experimental approaches to study tripartite interactions in the mycorrhizosphere. (**A**) Compartmented plate separates; a root compartment (RC) with ^13^C-enriched glucose and carrot root organ culture inoculated with *Rhizophagus irregularis* and a hyphal compartment (HC) only *R. irregularis* can access supplemented with ^15^NH_4_. (**B**) Compartmented plate that separates the RC from the HC. The HC is inoculated with phosphorus-solubilizing bacteria (PSB) and enriched with phytate. Red circles represent PSB colonies. (**C**) *Brachypodium distachyon* inoculated with *R. irregularis*. A micron mesh creates a root-free zone (RFZ). ^15^N-enriched organic matter is wrapped in the micron mesh. (**D**) Two-compartment growth box with a 1-mm air gap interface covered in a micron sheet. The plant compartment contains *Avena barbata* inoculated with *R. irregularis*. The air gap prevents ^18^O-enriched water transfer from the RFZ to RC, and the micron mesh prevents root growth from the RC to RFZ. (**E**) In growth, the core micron filter creates *B. distachyon* RFZ. DNA extractions performed in the root-free zone provided hyphal microbiome data. (**F**)The random forest model (RFM) and generalized linear model (GLM) filter out parameters that have less impact on the mycorrhizal growth response. (**G**) Extractions from different field-soil types are analyzed with co-occurrence networks to identify hub and/or keystone taxa; red lines represent negative interactions, green lines represent positive interactions, and black lines represent neutral interactions. Synthetic communities (SynComs) are designed from the results of such networks and are composed of hub/keystone taxa. (**H**) Field–rhizosphere communities are isolated and grown in liquid culture with metabolite profiles designed from root exudates of hydroponically grown plants. Right to left*:* salicylic acid, indole acetic acid, and nicotinic acid. Changes in the community composition were observed according to differences in the metabolite profile. Black arrows represent a potential perspective on how lab and field work can feed into each other. Figure is created using BioRender.

AMF can also acquire and transfer N to their host. Transfer of C from the host into AMF tissue directly induces N uptake and transport in the AMF, suggesting an “exchange rate” similar to that observed with P (43). AMF acquires and transfers exogenous N to the plant from different sources (44), including decomposed organic matter (27, 33, 45) and ammonium (26). Hestrin et al. tracked the flow of ^15^N, derived from labeled organic matter, through AMF hyphae into plant roots (Fig. 2C) (27). They also tracked the flow of ^13^C (27) and visualized photosynthate in hyphae and hyphal-associated bacterial decomposers, qualitatively showing the movement of C from plants to bacteria through AMF (27). Even if this transfer is passive (46, 47), it may change the value of N in tripartite–nutrient dynamics (48).

When N competition is considered, more microbial players become important. Bukovska et al. observed the suppression of specific bacterial communities, including ammonium oxidizers, in the presence of AMF (49). In contrast, protist populations were uninhibited. Based on this observation, Bukovska et al. proposed protist grazing of bacterial decomposers, and subsequent release of ammonium ions provided N for AMF without consequences to the protist. While this interaction needs more investigation, experiments that only consider plants, AMF, and N sources may exclude multiple kingdoms of taxa that are performing N-cycling processes. Since AMF cannot acquire C in the absence of plants or mobilize different forms of organic N (50, 51), AMF-related nutrient dynamics are influenced by C provided by the host plant and inorganic N released by soil microbes, regardless of any byproduct mutualism arising from microbial interactions (Fig. 1). The availability and exchange of organic forms of nutrients change the rules of previously identified nutrient exchanges between the plants, AMF, and the microbes associated with hyphae. Therefore, we must design our experiments using appropriate tools to reassess the rules governing these exchanges and underpinning the ecophysiology of AMF.

### Tools to unravel the tripartite network

We have relatively more information on the microbes present in the mycorrhizosphere (36, 47, 52–56) than on the processes performed by them. These interactions happen in close proximity to roots and extraradical hyphae, making it challenging to identify respective microbes involved in a specific nutrient cycling process. In this section, we will review how the research field is well-poised to achieve this identification through technology and creative methodology.

### Compartmented systems

Since AMF are obligate biotrophs, and mycorrhizosphere microbes live in close proximity to hyphae and roots, discerning between the tripartite member’s physiology is challenging. A simple and elegant approach to overcoming this concern of proximity is building compartmented systems that create root or microbe-free zones. Further addition of air gaps and micron mesh to these compartmented chambers can allow interaction of AMF with bacterial communities in root-free zones (RFZ), while still allowing for nutrient flow between the members. Various designs of the compartmented systems are being used, each tailored differently depending on the specific question being asked.

The simplest method to separate tripartite members is a compartmented petri plate. The plate’s raised wall separates two compartments containing different media (Fig. 2A and B). Only AMF hyphae in these systems can grow over the wall. The root compartment (RC) side of the plate contains carrot root organ cultures inoculated with AMF. The AMF grows over the wall, creating the hyphal compartment (HC), and a root-free zone (RFZ). Labeled nutrients or bacterial species can be added to the HC, and if the bacterially derived or labeled nutrient is found within the RC, it suggests AMF-dependent transport of the nutrient. These plates are effective at elucidating mechanisms, but the one-dimensional medium and lack of photosynthetic tissue limit our ability to extrapolate system processes to more realistic conditions.

Micron meshes can effectively isolate microbial interactions with hyphae from roots, hence avoiding the confoundment of cross-kingdom biology in more realistic settings. Hestrin et al. created an RFZ in a mesocosm by wrapping ^15^N-labeled organic material in a micron mesh (Fig. 2C) (27). This simple addition to the experiment prevented the roots from directly accessing this N source, suggesting N was transported out of the RFZ by AMF hyphae. Hence, the mesh becomes a powerful tool for separating roots and hyphae while tracking nutrient flow within the tripartite system. In another study, use of in-growth cores also created RFZs within the core, allowing for extra-hyphal microbiome assessment (Fig. 2E) (53). The core could be used to study the metabolome and transcriptome of the AMF–microbe interface. Untargeted mass spectrometry from a core could provide information on AMF exudates that may be important in recruiting other microbes. Hyphal transcriptomics within the core would investigate gene expression changes of only the extraradical mycelium of AMF, whereas core-metatranscriptomics could elucidate processes within the extra-hyphal microbiome. Thus, the in-growth cores provide a simple and cost-effective tool to propel our understanding of the tripartite system forward.

The addition of an air gap to an RFZ can further isolate the tripartite members. Kakouridis et al. created a mesocosm with two compartments separated by a 1-mm air gap that prevents water transfer between the compartments (57). Each connecting wall of the air gap had a micron mesh that created an RFZ (Fig. 2D). This allowed AMF to proliferate in both compartments, so if there were to be any intercompartmental water flow, it would have to be through hyphae. The authors then added H_2_^18^O in the RFZ and observed H_2_^18^O in the plant, suggesting AMF hyphae-mediated water transport. This combination of the air gap and micron mesh could prevent bacterial transfer between compartments, creating a bacteria-free zone (BFZ). The combination of the BFZ, RFZ, and heavy isotope techniques discussed in the next section would allow for controlled tracking of bacterial-derived nutrients and could help discern the partners responsible for transport of those nutrients. Overall, the compartmented chambers are useful in isolating different tripartite members and can be enhanced through the addition of well-established biochemical techniques. In the following section, we will explore how these biochemical techniques and experimental design can help identify the processes performed by the tripartite members.

### Listening among the noise

Soil is an inherently complex chemical matrix. Diverse mineral makeups, life forms, and organic materials create challenges due to adsorption, hydrological variability, ion exchange, pH variability, and more when characterizing nutrient processes in the soil. These challenges make techniques such as comparative mass-spectrometry (CMS) less useful on their own (58). However, combining CMS with heavy isotopes can track nutrient flow through the tripartite system with precision.

Different mass spectrometry approaches can be used with stable isotope tracking to identify how nutrients move throughout this system. Hestrin et al. used a well-established technique, isotope ratio mass-spectrometry (IRMS), to assess ^15^N levels in shoots (Fig. 2C) and nanoscale secondary ion mass-spectrometry (Nano-SIMS) with both ^13^C and ^15^N to visualize nutrient exchange within the tripartite system under different levels of soil N (27). Kakouridis et al. recorded transpiration and translated data with an isotopic mixing model to quantify H_2_^18^O transferred by AMF hyphae (Fig. 2D). Smith et al. showed the importance of fungi in the P-nutrient trade, which was independent of the plant growth response with P^33^ (59, 60). P isotopes’ radioactivity makes environmental application unsafe, so labeling PO_4_^−3^ with ^18^O has been attempted, but is limited by O transfer from PO_4_^−3^ to H_2_O in many biological processes (61). Tracking different stable isotopes can help elucidate mechanisms of nutrient dynamics driven by respective microbes precisely within the noisy environment of the mycorrhizosphere.

Stable isotope probing (SIP) can identify species receiving labeled nutrients (62). Followed by a pulse of ^13^CO_2_, researchers isolated microbes that assimilated heavy isotopes into their DNA through density gradient centrifugation. The sequencing of these fractions revealed metabolically active microbes (63). Recently, Nuccio et al. enhanced SIP with semi-automated, high-throughput sequencing (HT-SIP), where they identified AMF-associated taxa enriched in ^13^C post-^13^CO_2_ exposure (64). Combining this pipeline with compartmented mesocosms would allow HT-SIP sampling of different compartments to simultaneously answer who receives a nutrient and track its flow in the system.

The combination of stable isotope tracking and mechanical isolation enhanced the precision and accuracy of these experiments. Organic matter covered by a micron mesh ensured that the transfer of ^13^C to bacteria and ^15^N uptake was not directly from roots but through hyphae. The combination of the air gap and mesh also showed water transport through hyphae. Overall, these studies demonstrate the impact of simple tweaks in the experimental design.

### Deconstructing and reconstructing interactions

The practical application of these findings necessitates large-scale experiments. *In vitro* experiments provide mechanistic information about microbial activity in the mycorrhizosphere. However, species diversity and abiotic variables increase significantly in the field, leading to confounding results and interpretations. So, how can we overcome these complex experimental hurdles to make findings more relevant in a field context? Below, we review recent methods that can bridge the gap between *in vitro* and *in situ* experiments.

Statistical modeling can help find impactful variables within the myriad of field data. Lutz et al. used a combination of well-known methods to identify variables correlated with the mycorrhizal growth response (MGR) in AMF-inoculated fields (Fig. 2F) (65). They reduced soil parameters through pairwise correlation, which filtered out parameters that did not correlate with the MGR. They fed filtered parameters into a random forest model, a stepwise model, and an exhaustive model screening using “glmulti” (66) and found 15 parameters that correlated with MGR in each method. Furthermore, these 15 parameters were used as vectors in a principal component analysis with MGR values, plotted to assess parameter importance in creating the different MGR groups: high, medium, and low. The same technique was used with microbiome composition data to identify MGR-correlated taxa. This multimodal approach suggested correlations that needed to be assessed for predictive power. Lutz et al. used the correlated parameters in a generalized linear model and found microbial taxa that predicted the MGR more accurately than any other parameter. This approach narrowed down the number of variables that are important to consider when assessing the performance of the AMF inoculum in the field. Furthermore, it could improve *in vitro* experimentation by introducing only the most impactful variables and taxa from the field into a controlled setting, avoiding needless complexity while ensuring experiments are contextually relevant.

Co-occurrence network analyses identify core microbial members correlated with the stability and resilience of the soil microbiome (Fig. 2G) (67–70). These findings helped develop a consortium of microbes referred to as synthetic communities (SynComs) that can be used *in vitro* and in the field, providing important core community processes (Fig. 2G) (52, 56, 67, 68, 70–72). These SynComs can help bridge the gap between the sterile environment of the lab and the complex environment of the field and enable scientists to infer causal relationships in the mycorrhizosphere.

Exometabolomic assays combine field taxa and laboratory sterility to provide insights into more prominent chemical phenomena. The different members of the tripartite network excrete many metabolites that impact the mycorrhizosphere (42, 73). Zhalnina et al. collected root exudates at different plant growth stages and grew field-isolated bacteria in media supplemented with these root exudates to observe the effects of metabolites on the microbial community (Fig. 2H). This reductionist approach can help understand field-isolated microbial responses to certain metabolite profiles with fewer complications.

Taking the information gained through these modeling and metabolomic approaches to compartmentalized apparatus experiments would increase the confidence in extrapolating *in vitro* results to the field. We can assess how tripartite member processes influence community ecology by introducing treatments inoculated with a core SynCom. Additionally, we can simulate field conditions by treating soils in the compartmented chambers with metabolic profiles that closely resemble those found in fields. Using statistical correlations and models, we can ensure impactful variables are present in experiments without erroneous complications. Building upon this work, we can enrich *in vitro* experiments with *in situ* findings and vice versa.

### The importance of tripartite perspective

In this review, we highlighted research that suggests that emphasis is needed on all the kingdoms involved in the tripartite nutrient exchange. While P-related research is splintered between bipartite interactions, research focused on the exchange between all tripartite members will be valuable. In this study, we compiled the growing body of research that suggests that this tripartite exchange occurs in the “nitrogen market” as well. Furthermore, we must consider that P dynamics influence N dynamics and vice versa (12), and integration of N-related research with that of P is vital for accurate interpretation. Careful methodology can uncover the ecophysiological phenomena underpinning these exchanges. In this review, we presented a framework and the tools to piece together small portions of this tripartite to inform increasingly scalable research that will enhance our capabilities in increasing nutrient use efficiency by utilizing microbes residing in the mycorrhizosphere.
